# Multigene Panel Testing Yields High Rates of Clinically Actionable Variants Among Patients With Colorectal Cancer

**DOI:** 10.1200/PO.22.00517

**Published:** 2022-11-12

**Authors:** Sarah E. Coughlin, Brandie Heald, Dana Farengo Clark, Sarah M. Nielsen, Kathryn E. Hatchell, Edward D. Esplin, Bryson W. Katona

**Affiliations:** ^1^ University of Pennsylvania Perelman School of Medicine, Philadelphia, PA; ^2^ Invitae, San Francisco, CA

## Abstract

Whether germline multigene panel testing (MGPT) should be performed in all individuals with colorectal cancer (CRC) remains uncertain. Therefore, we aimed to determine the yield and potential clinical impact of MGPT across a large, diverse CRC cohort.

**METHODS:**

This was a retrospective cohort study of adults with CRC who underwent MGPT of > 10 genes at a commercial laboratory between March 2015 and May 2021. All data were prospectively collected through a single commercial laboratory and retrospectively analyzed.

**RESULTS:**

A total of 34,244 individuals with a history of CRC underwent germline MPGT and were included in the analysis. This cohort was predominantly female (60.7%), White (70.6%), and age 50 years or older (68.9%), with 35.5% also reporting a noncolorectal malignancy. At least one pathogenic/likely pathogenic germline variant (PGV) was found in 4,864 (14.2%), with 3,111 (9.1%) having a PGV associated with increased CRC/polyposis risk and 1,048 (3.1%) having an otherwise clinically actionable PGV. Larger gene panels were not clearly associated with higher yield of clinically actionable PGVs. PGVs were more prevalent in individuals of Ashkenazi Jewish descent (*P* < .001) and Hispanic ethnicity (*P* < .001). Across all ages, panel sizes, and races/ethnicities, the rate of clinically actionable PGVs on MGPT was 7.9% or greater. A variant of uncertain significance was identified in 13,094 individuals (38.2%). Identification of a variant of uncertain significance associated with panel size (*P* < .001) and was lower in individuals of Ashkenazi Jewish descent (*P* < .001), but higher in Black, Asian, and Hispanic individuals (*P* < .001).

**CONCLUSION:**

To our knowledge, this is the largest study to date examining MGPT in CRC, demonstrating high rates of clinically actionable variants detected across all age groups, panel sizes, and racial/ethnic groups. This work supports consideration of broadening germline genetic testing criteria for individuals with CRC.

## INTRODUCTION

Germline genetic testing is being increasingly used to assess for hereditary cancer predisposition and is most often performed using multigene panel testing (MGPT).^1^ Identification of pathogenic or likely pathogenic germline variants (PGVs) in hereditary cancer predisposition genes can have significant medical management implications for both patients and their families.^2,3^ For individuals with cancer, guidelines governing MGPT often depend on the primary tumor type. For ovarian cancer, pancreatic adenocarcinoma, and high-risk and/or metastatic prostate cancer, MGPT is recommended for all affected individuals.^4,5^ There has also been momentum toward recommending universal MGPT for breast cancer.^6^ However, for other cancer types, such as colorectal cancer (CRC), recommendations for performing MGPT are more restrictive.^2,3^

**CONTEXT**

**Key Objective**
Germline genetic testing for colorectal cancer (CRC) is currently recommended for only a subset of patients with CRC who meet certain high-risk criteria. Whether universal germline multigene panel testing (MGPT) should be performed in all individuals with CRC remains uncertain. Therefore, we performed a retrospective cohort study of 34,244 individuals with CRC who underwent MGPT to determine the yield and potential clinical impact of MGPT across the largest and most diverse cohort of patients with CRC to date.
**Knowledge Generated**
We found that 14.2% of patients with CRC carried at least one pathogenic/likely pathogenic germline variant (PGV). Clinically actionable PGVs were identified in 11.9%, including 9.1% with a PGV in a gene associated with CRC/polyposis risk, and these rates remain high regardless of the age of testing, number of genes included on the panel, and across all races/ethnicities.
**Relevance**
Together, these findings provide intriguing new data supporting the broadening of eligibility criteria for MGPT among patients with CRC.

CRC is one of the most common causes of cancer in both men and women.^7^ Although Lynch syndrome, resulting from a PGV in *MLH1*, *MSH2*/*EPCAM*, *MSH6*, or *PMS2*, remains the most common hereditary cause of CRC, there have also been an increasing number of other genes associated with elevated CRC risk.^8-11^ Recent guidelines recommend MGPT for individuals with CRC only under certain circumstances, including an age of diagnosis younger than 50 years, having a synchronous or metachronous Lynch syndrome–related cancer, having a family history of certain Lynch syndrome–related cancer(s), and if there are abnormalities in mismatch repair immunohistochemistry (MMR IHC) of the CRC.^2^ However, the majority of patients with CRC do not fulfill these testing criteria and, therefore, may not be offered nor receive insurance coverage for MGPT.^9^ Established criteria for MGPT in CRC will also invariably miss a portion of patients who harbor a PGV in a cancer risk gene.^10,11^ Universal tumor screening with MMR IHC would miss 39% of these PGVs^12^ while germline MGPT among all patients with solid tumors led to identification of clinically actionable findings in 6% of patients that would have not been identified using guideline-based criteria for genetic testing.^10^ In response to these concerns, the recently updated NCCN guidelines do not recommend, but do allow for, consideration of germline MGPT in all individuals with CRC.^3^

Several small studies have assessed the yield of MGPT in patients with CRC, with high rates of actionable findings identified. A single institution study from the United States of 1,058 individuals with CRC who underwent MGPT with a 25-gene panel showed that 10% had a PGV in a cancer susceptibility gene.^13^ Another study of 361 patients with CRC who underwent MGPT with an 80-gene panel at three US centers found PGVs in 16% of patients.^11^ Other studies of similar size performed at single centers showed a similar yield of PGVs in cancer risk genes.^14,15^ Although these studies show high rates of PGVs in individuals with CRC, they were performed in tertiary care centers with likely ascertainment and referral bias and, therefore, may not reflect more racially/ethnically and geographically diverse populations. Additionally, these studies do not look at the yield of MGPT among different panel sizes.

In this study, we examine the largest and most diverse sample set-to-date of patients with a history of CRC who underwent MGPT at a commercial laboratory. We highlight the frequency of clinically actionable PGVs identified and stratify these data based on age, number of genes tested, and clinician-reported race/ethnicity.

## METHODS

### Study Design and Patient Population

We conducted a retrospective cohort study of all individuals with a CRC diagnosis who underwent MGPT of more than 10 genes at a commercial laboratory between March 2015 and May 2021. We collected patient demographics from test requisition forms and results of germline DNA sequencing. Patients tested for 10 or fewer genes and individuals with possible mosaic PGV results (defined as a variant that is not present at an allelic fraction that is consistent with or expected in diploid or heterozygous situations) were excluded from the analysis. Patient demographics include age at testing, sex, clinician-reported race/ethnicity, and diagnosis of noncolorectal malignancies. The primary outcome was identification of a PGV associated with cancer predisposition. Variant of uncertain significance (VUS) identification was evaluated as a secondary outcome.

### Sequencing

Full-gene sequencing, deletion and duplication analysis, and variant interpretation were performed at Invitae, as previously described.^16^ Briefly, next-generation sequencing was performed on genomic DNA isolated from patient samples to at least 350× average coverage of 2 × 150 reads, with a minimum of 50× required at every targeted position. Copy-number variants were called using read-depth analysis with CNVitae. Variants were interrogated and classified using refined American College of Medical Genetics and Genomics criteria.^17^ PGVs were orthogonally confirmed in accordance with Invitae standard operating practices.^18^

### Variant Interpretation

PGVs were categorized by clinical relevance (Data Supplement). Clinically actionable PGVs were defined as both PGVs in genes with reported CRC or polyposis risk (CRC/polyposis) and PGVs in other actionable genes associated with published management recommendations and/or potential therapeutic implications (other actionable). PGVs without associated clinical management or therapeutic implications were defined as nonactionable.

### Statistical Analysis

All variables of interest were either binary or categorical and reported as proportions and counts. Pearson χ^2^ tests were used to estimate *P* values comparing categorical variables. Exploratory univariate and multivariate logistic regression analyses were additionally performed to identify factors predictive of testing positive for a PGV and for a VUS. A *P* value < .05 was considered statistically significant for these tests. All analyses were performed using Stata/IC 15.0 or RStudio statistical programs. The results from an initial preliminary analysis of this data set were previously presented in abstract form, with the current analysis being further refined and thus representing updated data.^19^

## RESULTS

A total of 36,647 individuals with a reported history of CRC underwent germline MPGT during the study period. Excluded individuals included 2,291 who underwent testing for 10 or fewer genes and 112 with a potential mosaic PGV result. Therefore, 34,244 individuals met inclusion criteria (Table 1) and were included in the analysis. These individuals were primarily female (60.7%), White (70.6%), and 50 years or older (68.9%). The number of genes tested ranged from 11 to > 80, with MGPT of more than 80 genes performed in 29.4%. A noncolorectal primary malignancy was reported in 35.5%; the most common of which was breast cancer in 13.8%.

**TABLE 1.**
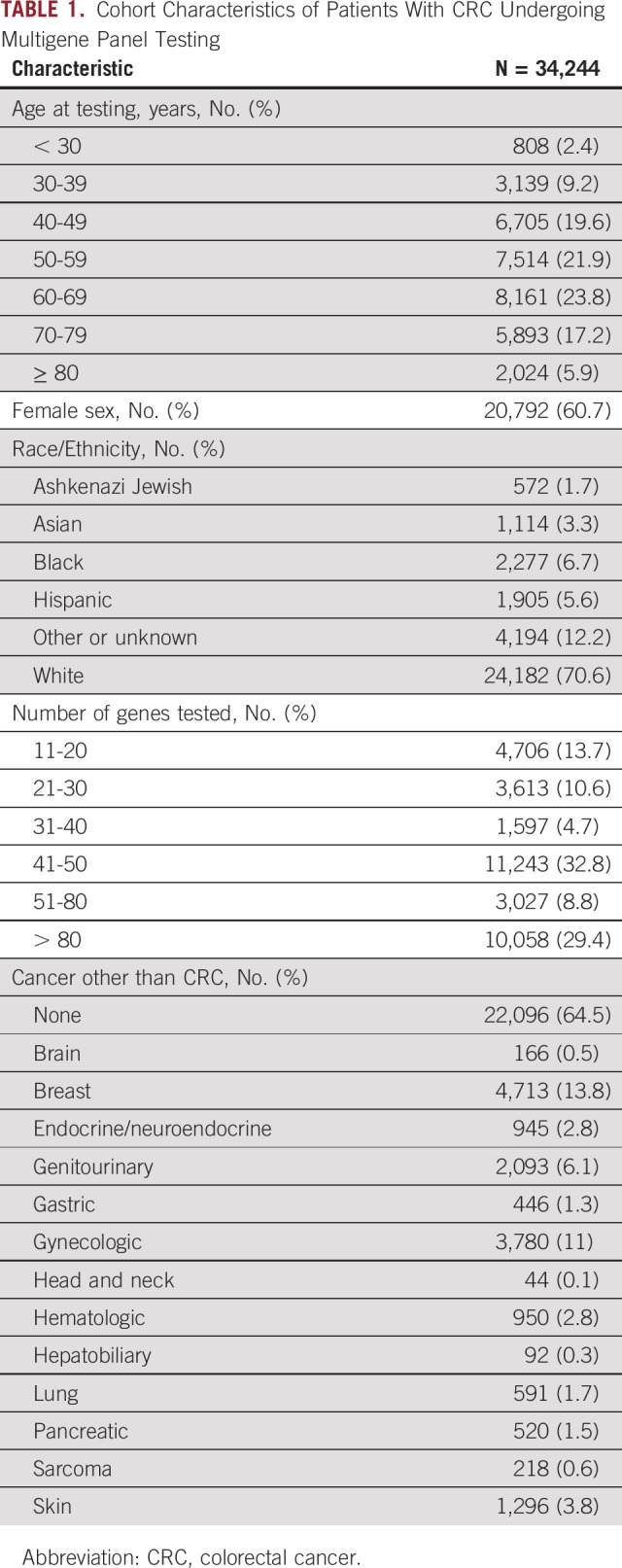
Cohort Characteristics of Patients With CRC Undergoing Multigene Panel Testing

**FIG 1.**
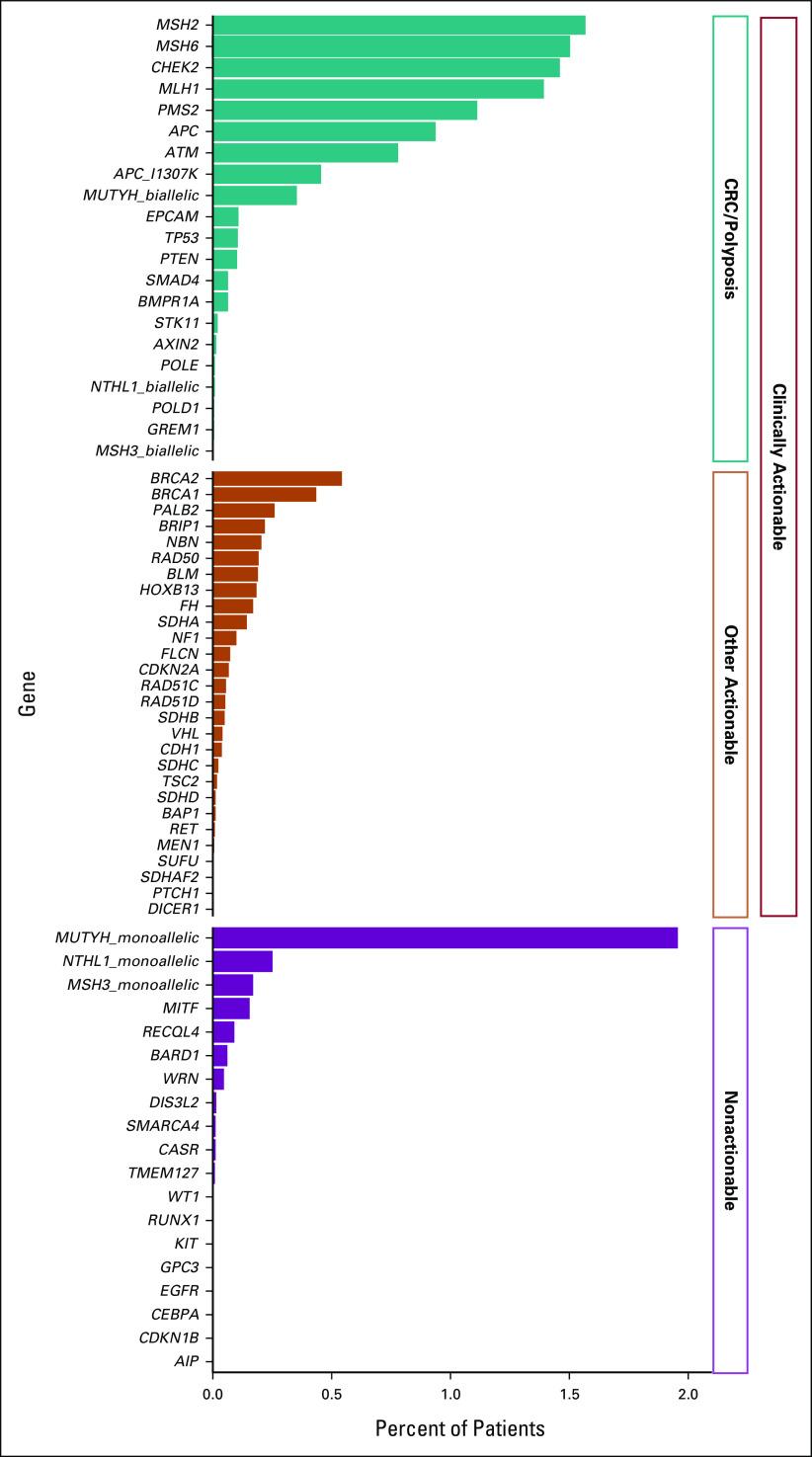
Percent of patients with PGVs by individual gene and clinical relevance. Genes with at least one PGV identified are pictured. CRC, colorectal cancer; PGV, pathogenic germline variant.

There were 4,864 (14.2%) individuals who carried at least one PGV (Table 2), with more than one PGV identified in 462 (1.4%). A total of 4,059 (11.9%) individuals carried a clinically actionable variant, including 3,111 (9.1%) carrying a PGV in a gene associated with an increased CRC/polyposis risk. Lynch syndrome–related PGVs were identified in 1,945 (5.7%) individuals; 477 (1.4%) carried a PGV variant in *MLH1*, 537 (1.6%) in *MSH2*, 513 (1.5%) in *MSH6*, 381 (1.1%) in *PMS2*, and 37 (0.1%) in *EPCAM* (Fig 1, Data Supplement). Other actionable PGVs were identified in 1,048 (3.1%) individuals. The three most prevalent other actionable genes in which a PGV was identified were *BRCA2* (187, 0.6%), *BRCA1* (149, 0.4%), and *PALB2* (89, 0.3%). A nonactionable PGV was identified in 952 (2.8%), including 670 individuals (2.0%) carrying a monoallelic *MUTYH* variant.

**TABLE 2.**
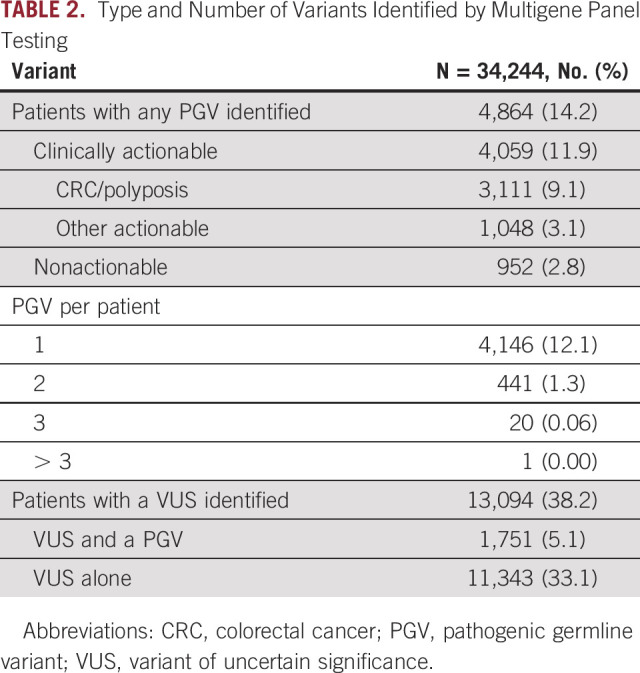
Type and Number of Variants Identified by Multigene Panel Testing

**FIG 2.**
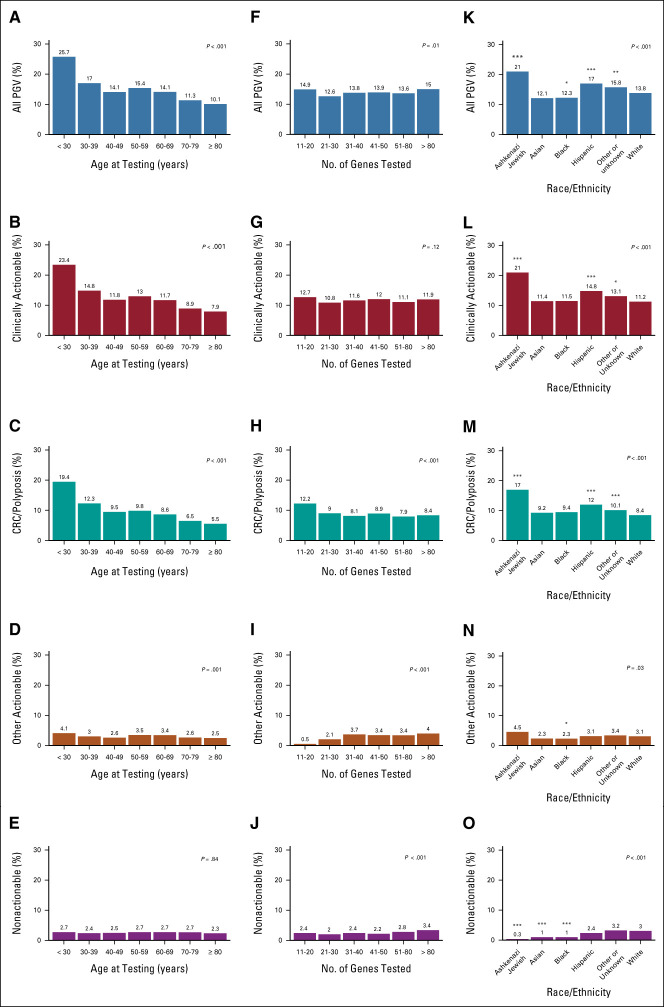
MGPT results by patient demographics: (A) distribution of all PGVs, (B) clinically actionable PGVs, (C) CRC/polyposis PGVs, (D) other actionable PGVs, (E) nonactionable PGVs by age; (F) distribution of all PGVs, (G) clinically actionable PGVs, (H) CRC/polyposis PGVs, (I) other actionable PGVs, (J) nonactionable PGVs by number of genes tested; (K) distribution of all PGVs, (L) clinically actionable PGVs, (M) CRC/polyposis PGVs, (N) other actionable PGVs, (O) non-actionable PGVs by race and ethnicity. Clinically actionable PGVs include CRC/polyposis and other actionable PGVs. ***P < .001; **P < .01, *P < .05. CRC, colorectal cancer; MGPT, multigene panel testing; PGV, pathogenic germline variant.

The prevalence of a PGV was highest among younger individuals but remained over 10% across all age groups (Fig 2A). Among patients age younger than 30 years when tested, 25.7% had a PGV compared with 17% at age 30-39 years, 14.1% at 40-49, 15.4% at 50-59, 14.1% at 60-69, 11.3% at 70-79, and 10.1% at 80 years or older (*P* < .001; Fig 2A, Data Supplement). Individuals tested at an age younger than 30 years had a clinically actionable variant found in 23.4% compared with 14.8% at age 30-39 years, 11.8% at 40-49, 13.0% at 50-59, 11.7% at 60-69, 8.9% at 70-79, and 7.9% age older than 80 years (*P* < .001; Fig 2B, Data Supplement). No more than 2.7% of any age group tested were found to have a nonactionable PGV (Fig 2E).

Gene panel size was associated with yield of PGV (*P* = .01; Fig 2F, Data Supplement). Larger gene panels were not associated with higher yield of clinically actionable variants (*P* = .12; Fig 2G). Subset analysis of CRC/polyposis genes demonstrates a higher yield among individuals in whom 11-20 genes were tested as compared with those with whom broader MPGT was performed (*P* < .001; Fig 2H). By contrast, larger panels were associated with a higher yield of other actionable and nonactionable variants (*P* < .001; Figs 2I and 2J).

Compared with White individuals, who comprised the majority of the cohort, PGVs were more prevalent in individuals of Ashkenazi Jewish descent (*P* < .001) and those identified as Hispanic (*P* < .001; Fig 2K, Data Supplement). The same was true for clinically actionable and CRC/polyposis-associated variants (Figs 2L and 2M).

A total of 12,148 individuals reported another cancer diagnosis in addition to CRC (Data Supplement). A PGV was found in 14.8% of individuals with a second, non-CRC diagnosis compared with 13.9% of those without a second primary malignancy (*P* = .03). This difference was primarily driven by other actionable variants rather than CRC/polyposis or nonactionable variants; 3.9% of individuals with CRC and a non-CRC malignancy carried an other actionable variant as compared with 2.6% of patients with CRC alone (*P* < .001).

**FIG 3.**
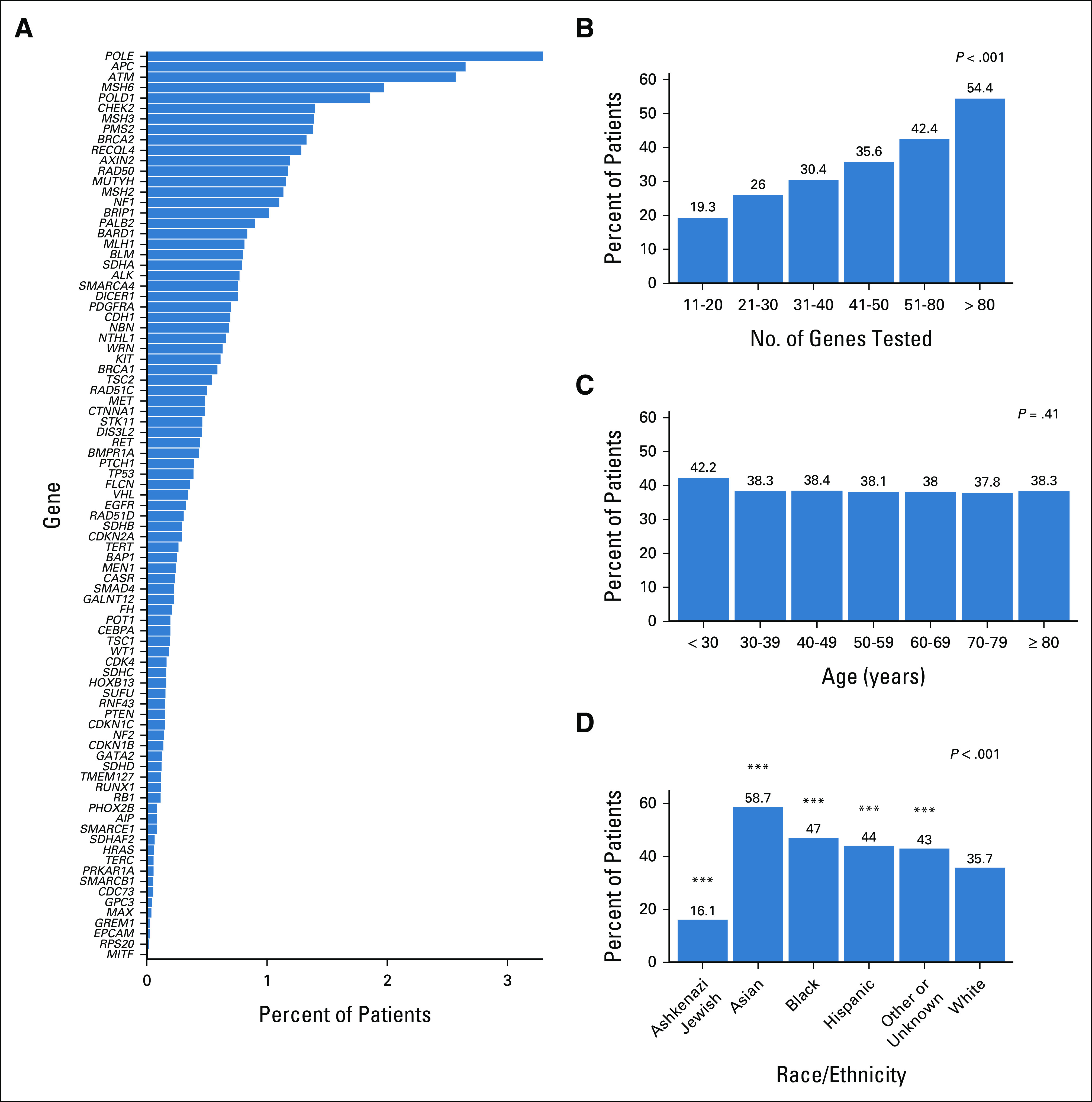
VUSs resulting from MGPT of patients with CRC. Percent of patients with (A) a VUS by individual gene, (B) number of genes tested, and (C) distribution of VUSs by age group; (D) VUSs by reported race and ethnicity compared with White individuals who comprised the majority of the cohort. ***P < .001. CRC, colorectal cancer; MGPT, multigene panel testing; VUS, variant of uncertain significance.

A VUS was identified in 13,094 (38.2%) individuals (Table 2). A VUS along with a PGV was found in 1,751 (5.1%) individuals while 11,343 (33.1%) carried a VUS alone. The three most prevalent genes in which a VUS was detected were *POLE* (1,129, 3.3%), *APC* (908, 2.7%), and *ATM* (880, 2.6%; Fig 3A and Data Supplement). In contrast to PGVs, VUSs were identified more frequently in larger panels (*P* < .001; Fig 3B). At least one VUS was identified in 19.3% of individuals in whom 11-20 genes were tested as compared with 26% of individuals with 21-30 genes tested, 30.4% with 31-40 genes tested, 35.6% with 41-50 genes tested, 42.4% with 51-80 genes tested, and 54.4% of individuals with more than 80 genes tested. VUS results were not associated with age at testing (*P* = .41; Fig 3C) but were significantly associated with race and ethnicity (Fig 3D). A VUS was less frequently identified in individuals of Ashkenazi Jewish descent compared with those identified as White (*P* < .001), whereas a VUS was more frequently identified in individuals identified as Black (*P* < .001), Asian (*P* < .001), or Hispanic (*P* < .001) compared with White individuals.

Univariate analysis supports age at testing (odds ratio [OR], 0.90; *P* < .001; 95% CI, 0.88 to 0.91) and race/ethnicity (OR, 1.03; *P* < .001; 95% CI, 1.02 to 1.05) as predictors of having a PGV (Data Supplement). Those individuals tested in any age group older than age 30 years were less likely to carry a PGV compared with those younger than 30 years (*P* < .001). Compared with White individuals, those of Ashkenazi Jewish descent (OR, 1.60; *P* < .001; 95% CI, 1.31 to 1.95) and Hispanic ethnicity (OR, 1.28; *P* < .001; 95% CI, 1.13 to 1.44) were more likely to carry a PGV. The number of genes tested was weakly predictive of PGV status (OR, 1.02; *P* = .02; 95% CI, 1.00 to 1.04); subset analysis suggests that this is driven primarily by higher yield among those tested for 10-20 genes compared with broader MGPT. By contrast, the number of genes tested (OR, 1.37; *P* < ;.001; 95% CI, 1.35 to 1.39) and race/ethnicity (OR, 1.08; *P* < .001; 95% CI, 1.07 to 1.09), but not age at testing (OR, 0.99; *P* = .13; 95% CI, 0.98 to 1.00), were predictive of finding a VUS. Multivariate analyses adjusting for age, number of genes tested, and race/ethnicity support the same relationships for both PGV and VUS (Data Supplement).

## DISCUSSION

MGPT is increasingly being used for cancer risk assessment, with identification of PGVs being critically important for clinical management of affected patients and their families. Although MGPT is currently recommended for patients with CRC meeting defined high-risk criteria, limiting testing to these individuals misses PGVs in a subgroup of patients with CRC, leading to the continued debate about whether genetic testing criteria for patients with CRC should be broadened. Herein, we report on MGPT outcomes in a large cohort of patients with CRC who underwent MGPT at a commercial testing laboratory. Our data illustrate a high rate of PGVs identified in clinically actionable genes in this largest and most diverse CRC cohort reported on to date. These rates remain high regardless of the age of testing, number of genes included on the panel, and across all races/ethnicities. Together, these findings provide intriguing new data supporting the broadening of eligibility criteria for MGPT in patients with CRC.

Given the increased access to and decreasing cost of MGPT, having a yield of PGVs > 5% is widely considered a reasonable threshold whereby MGPT should be recommended.^20,21^ Clinically actionable variants were identified in 11.9% of patients with CRC in our study, including 9.1% with a PGV in a gene associated with CRC/polyposis risk. When stratifying these data by age at the time of testing, number of genes tested, and race/ethnicity, all subgroups with CRC consistently have a rate of clinically actionable variants > 5%. Furthermore, when examining the subgroup of genes associated with CRC/polyposis risk, the rate of identifying a PGV also remarkably remained > 5%, regardless of the age at time of testing, number of genes tested, and race/ethnicity.

Using a MGPT approach enables identification of PGVs in other cancer risk genes that are not classically associated with CRC and/or polyposis risk. In our cohort, 3.1% of patients were identified as having a PGV in a clinically actionable gene that does not currently have a known CRC or polyposis risk. Although these genes currently do not have a well-established CRC risk association, it is possible that such an association may be recognized in the future as more evidence is collected. Furthermore, identifying other non-CRC/polyposis associated clinically actionable genes, such as PGVs in *BRCA1* or *BRCA2* which were identified in 0.44% and 0.55% of our cohort, respectively, confer potential eligibility for PARP inhibitor clinical treatment trials and can have substantial, and possibly lifesaving, implications for cancer risk management for both patients and their families.

Given that CRC affects individuals of all sexes, races, and ethnicities, having MGPT data among a diverse cohort of patients with CRC is important. Our study population had diversity related to age at the time of testing, sex, race/ethnicity, and number of genes tested. With respect to age, patients were tested over a broad range of ages including more than 30% of whom were tested before age 50 years. Additionally, 60.7% of our cohort were female, and only 70.6% of our cohort were White, demonstrating that there was more racial and ethnic diversity in our cohort compared with other published series.^11-13^ Furthermore, using all patients tested at a commercial laboratory provides a more diverse sample set compared with studies that rely on data from a single or small group of centers.^11-14^ Finally, our data uses a diversity of panel sizes and shows that smaller, more-focused panels produce a similar yield of PGVs compared with larger panels.

Identifying VUSs on MGPT is inevitable, and our data show that as expected the frequency of VUS identification increases with panel size, with 54.4% of patients having a VUS identified with a gene panel of more than 80 genes. We also show substantial differences in VUS rate based on race/ethnicity, with all races/ethnicities other than individuals identifying as Ashkenazi Jewish, having a significantly higher rate of VUS compared with individuals identifying as White. Although it is likely that the VUS rate in non-White populations will decrease over time as more non-White individuals undergo genetic testing, recognizing these increased VUS rates across different races and ethnicities is important to allow for appropriate pretest counseling to ensure optimal expectations are set. Although differences in VUS rate are important to recognize in light of persisting concerns regarding medical mismanagement, recent studies suggest this concern may not bear out in practice.^22-24^

Although our data may suggest that eligibility for MGPT among patients with CRC should be broadened, it is also important to consider potential downsides that may result from doing so. Currently there are more than one million CRC survivors in the United States, and the majority of these individuals have not undergone MGPT.^25^ Therefore, any expansion of genetic testing criteria for patients with CRC would not only have to include prospectively identified CRCs but would also have to be applied to the large CRC survivor community. Such a feat may be difficult because of the current limited supply of genetic counselors^26^ and carries the risk of exacerbating already existing disparities in delivery of genetics services.^27^ The cost of performing MGPT in more patients with CRC would also need to be considered, as well as the potential psychosocial effects of MGPT.^28,29^ However, it should be noted that MGPT is underutilized in other cancer types with existing recommendations for universal testing, such as ovarian and metastatic prostate cancer, suggesting that there are also opportunities to optimize care delivery and overcome perceived barriers to universal germline testing for patients with CRC.

Finally, universal MMR-IHC, which should be performed routinely on all CRCs, has been an important and effective mechanism for identifying individuals who may have Lynch syndrome.^12^ While broadening eligibility for MGPT in CRC would obviate the need for MMR-IHC testing for Lynch syndrome screening, performing MMR-IHC or microsatellite instability testing on CRCs would still need to be performed for therapeutic decision making, including immunotherapy eligibility.^30^

Limitations of these data include that information used was from test requisition forms that were submitted by clinicians, and, therefore, confirmation of clinical information with the patients' primary medical record was not possible. Age reported was the age at the time of testing, and may be substantially older than when patients developed CRC. Additionally, our data included all individuals who had a diagnosis of CRC, even if the CRC was not the primary reason for these patients to undergo genetic testing. Finally, the patients included in the cohort studied had MPGT ordered under recent guidelines requiring a diagnosis of CRC younger than age 50 years or other features known to bias for the presence of germline genetic drivers.

Use of MGPT is a powerful tool that can identify clinically actionable PGVs in cancer risk genes. Herein, we provide MGPT data from the largest cohort to date of patients with CRC, where we show that there are high rates of clinically actionable variants among patients with CRC, independent of the age at the time of testing, the number of genes on the panel, and race/ethnicity. Together, these data provide evidence to support broadening of genetic testing criteria for patients with CRC.
